# High-Resolution Quantitative Mapping of Macaque Cervicovaginal Epithelial Thickness: Implications for Mucosal Vaccine Delivery

**DOI:** 10.3389/fimmu.2021.660524

**Published:** 2021-06-28

**Authors:** Kathleen L. Vincent, Patrice A. Frost, Massoud Motamedi, Edward J. Dick, Jingna Wei, Jinping Yang, Robert White, Marie-Claire Gauduin

**Affiliations:** ^1^ Department of Obstetrics and Gynecology, The University of Texas Medical Branch at Galveston, Galveston, TX, United States; ^2^ Population Health Program, Texas Biomedical Research Institute, San Antonio, TX, United States; ^3^ Southwest National Primate Research Center, San Antonio, TX, United States; ^4^ Department of Ophthalmology and Visual Sciences, The University of Texas Medical Branch at Galveston, Galveston, TX, United States; ^5^ Disease Intervention and Prevention Program, Texas Biomedical Research Institute, San Antonio, TX, United States

**Keywords:** high-resolution optical coherence tomography imaging, depo medroxyprogesterone, intravaginal, mucosal, rhesus macaque, vaccine delivery, cervical epithelial thickness, nonoxynol-9

## Abstract

Vaginal mucosal surfaces naturally offer some protection against sexually transmitted infections (STIs) including Human Immunodeficiency Virus-1, however topical preventative medications or vaccine designed to boost local immune responses can further enhance this protection. We previously developed a novel mucosal vaccine strategy using viral vectors integrated into mouse dermal epithelium to induce virus-specific humoral and cellular immune responses at the site of exposure. Since vaccine integration occurs at the site of cell replication (basal layer 100-400 micrometers below the surface), temporal epithelial thinning during vaccine application, confirmed with high resolution imaging, is desirable. In this study, strategies for vaginal mucosal thinning were evaluated noninvasively using optical coherence tomography (OCT) to map reproductive tract epithelial thickness (ET) in macaques to optimize basal layer access in preparation for future effective intravaginal mucosal vaccination studies. Twelve adolescent female rhesus macaques (5-7kg) were randomly assigned to interventions to induce vaginal mucosal thinning, including cytobrush mechanical abrasion, the chemical surfactant spermicide nonoxynol-9 (N9), the hormonal contraceptive depomedroxyprogesterone acetate (DMPA), or no intervention. Macaques were evaluated at baseline and after interventions using colposcopy, vaginal biopsies, and OCT imaging, which allowed for real-time *in vivo* visualization and measurement of ET of the mid-vagina, fornices, and cervix. P value ≤0.05 was considered significant. Colposcopy findings included pink, rugated tissue with variable degrees of white-tipped, thickened epithelium. Baseline ET of the fornices was thinner than the cervix and vagina (p<0.05), and mensing macaques had thinner ET at all sites (p<0.001). ET was decreased 1 month after DMPA (p<0.05) in all sites, immediately after mechanical abrasion (p<0.05) in the fornix and cervix, and after two doses of 4% N9 (1.25ml) applied over 14 hrs in the fornix only (p<0.001). Histological assessment of biopsied samples confirmed OCT findings. In summary, OCT imaging allowed for real time assessment of macaque vaginal ET. While varying degrees of thinning were observed after the interventions, limitations with each were noted. ET decreased naturally during menses, which may provide an ideal opportunity for accessing the targeted vaginal mucosal basal layers to achieve the optimum epithelial thickness for intravaginal mucosal vaccination.

## Introduction

The vaginal surface epithelium is a primary portal of entry for all sexually transmissible diseases including the Human Immunodeficiency Virus -1 (HIV-1). The vagina serves as a potential site of treatment, prevention, and vaccine administration (1–4); however, approaches to boost local immune responses and block entry of micro-organisms remain challenging. Oral pre-exposure prophylaxis (PrEP) has achieved success in preventing HIV with an efficacy of 99-100% in men who have sex with men (5) but has been less effective in heterosexual women with just 88-90% reduction in infections (6), a difference that has been attributed to lower drug concentrations in the vagina with the use of oral PrEP. Intravaginal PrEP treatments are designed to address this limitation by the administration of medication at the site of infection to prevent the transmission of sexually transmitted infections (STI) and HIV. However, well-established barriers associated with the use of oral or vaginal PrEP by women include stigma, cost, and adherence to the requirements of frequent use. These are some of the factors driving the interest for alternative strategies to prevent sexual infection transmission at the vagina, the primary site of viral entry in women, especially long acting prevention strategies that include medications or vaccines.

Recently, we reported on the development of a novel vaccine approach based on the ability of lentiviral vectors integrated into epithelial stem cells to induce specific cellular immune responses (3, 7). The use of a terminally differentiated epithelial cell promoter that drive expression of antigen constructs leading to viral protein production in the upper layers of the epithelium is central to the success of this approach. Our results showed that when administered intradermally to mice, the vaccine was expressed in the superficial epithelial cells layers and, although transduced cells were very low in number, high and sustained antibody production was observed *in vivo*. With the goal of delivering the vaccine directly to the site at risk of infection, the next step in vaccine development will be to evaluate this vaccine strategy through intravaginal administration. However, before conducting these studies, we must address the main challenge of this approach, which is accessing the replicating basal cells of the cervicovaginal epithelial cell layer. This type of epithelium-based vaccine inoculation may require a slight disruption or removal of the vaginal superficial cell layers to provide better access to the replicating basal layer, which is located 100-400 micrometers below the mucosal surface. A method for controlled removal or thinning of the surface epithelium is needed to ensure proper access of the vaccine to the basal layer (8).

The female Rhesus macaque (*Macaca mulatta*) model was used in this study, as it has been found to be the best model for vaccine development and microbicide efficacy testing due to similarities to the human vaginal tract and immunologic response (9). Various methods have been employed to thin the vaginal epithelial layers and improve the accessibility of the basal layers, however the extent and consistency of induced thinning in Rhesus macaques has not been well documented. Gentle abrasions have been used to alter the epithelial layers to facilitate Herpes Simplex Virus (HSV)-2 genital infection and to induce HSV recurrence in skin in mice (10, 11). Light abrasions have also been reported in cynomolgus macaques using a cytobrush to access the vaginal epithelial prior vaccination with genital human papillomavirus (HPV) (12). Nonoxynol-9 (N9), a chemical surfactant spermicide, has been shown to thin the vaginal epithelium after a single dose in sheep and after multiple doses in humans and macaques through surface cell layer disruption (13–15). Depomedroxyprogesterone acetate (DMPA) has been used in microbicide studies to thin the vaginal epithelial layers and facilitate viral infection with simian immunodeficiency virus (SIV) or simian-human immunodeficiency virus (SHIV) in nonhuman primate, or with HSV virus in mice (11, 16). Being able to determine the degree of thinning in real time is important for determining timing and location for delivery of a mucosal vaccine, therefore a method for quantifying epithelial thickness is needed.

Observations of epithelial thickness (ET) are not quantifiable with conventional colposcopy, as it does not have the resolution or in-depth capability to obtain these measurements. Biopsies allow for quantification of epithelial thickness, however, measurements cannot be obtained in real time due to the need for processing, sectioning, and staining the tissue. ET measurements from a biopsy provide only localized details about thickness at the site of biopsy, which can be misleading when the epithelium is heterogeneous. Taking a biopsy alters the tissue and can provide a portal of entry for sexually transmitted pathogens, an undesirable effect when the goal is to protect against infections (16, 17). Noninvasive real-time imaging technology overcomes the limitations of colposcopy and biopsy. High resolution imaging with optical coherence tomography (OCT) has been applied in sheep, pigtailed macaques, and humans for measuring vaginal epithelial thickness (14, 15, 18). Prior OCT imaging of the vaginal mucosa has demonstrated vaginal epithelial thickness reduction after mechanical abrasion and treatment with vaginal products that strip the superficial layers of the epithelium (14, 15, 18). OCT, similar to ultrasound except that it uses light instead of sound waves, provides an in-depth “optical biopsy” of the mucosa. ET estimations can be made *in vivo* in real time using the measure bars adjacent to the image, while precise measurements can be obtained after exporting the images and using image processing software. Additionally, images can be obtained longitudinally and repeatedly without damaging the epithelium, allowing for evaluation of multiple sites within the vagina.

An ideal approach to thinning the epithelium for mucosal vaccine inoculation would include inducing epithelial thinning without bleeding and without disruption of the epithelial surface or exposure of the lamina propria. Additionally, such method would facilitate the delivery of the vaccine by better exposing the epithelium basal layers without interfering with the natural growth of the epithelium stem cells and development of mucosal immune responses. Consistent thinning across the mucosal surface would allow better surface area in contact with the vaccine inoculation. In this study, we evaluated mechanical (cytobrush abrasion), chemical (N9), and hormonal (DMPA) methods to modify the thickness of the cervicovaginal epithelium as a means to access the basal layers. Optical coherence tomography was used before and after treatment to map each female macaque’s cervical and vaginal epithelial thickness (ET). OCT findings, confirmed by biopsies, were key to evaluate our methods for epithelial thinning by visualization and quantification of cervicovaginal epithelial thickness at the intended site for mucosal vaccine inoculation.

## Materials and Methods

### Experimental Animals

A total of twelve naïve uninfected juvenile (early-pubertal) Indian-origin female Rhesus macaques (*Macaca mulatta*) that had never been sexually active, 3.8 to 4.8 years of age and weighing 5 to 7 kg, were used for this study. Animals were obtained from the Southwest National Primate Research Center (SNPRC) specific pathogen free (SPF) colony (seronegative for HIV-2, SIV, type D retrovirus, and simian T-cell lymphotropic virus type 1). Animals were housed in accordance with the American Association for Accreditation of Laboratory Animal Care Standards regulations. The Institutional Animal Care and Use Committee of the Texas Biomedical Research Institute approved the animal protocol (IACUC 1387MM, 04/05/2013).

### Study Design

The female macaques (n=12) were assigned to one of four treatment groups, with 3 animals per group: control group (Group 1), mechanical treatment group with cytobrush abrasion (Group 2), chemical treatment group with intravaginal N9 (Group 3), or hormonal treatment group with intra-muscular DMPA (Group 4) (**Table 1**). The untreated control group (Group 1) did not receive any treatment to thin the epithelial layer. Group 2, the mechanical treatment group, had abrasions to the vagina and posterior fornix created with a cytobrush. Group 3, the chemical treatment group, was given 2 intravaginal doses of 4% N9 (Conceptrol, Ortho Pharmaceuticals) 14 hours apart. A stainless steel avian feeding tube was affixed to a 3cc syringe for administering the intravaginal treatment. Group 4, the hormonal treatment group, was given 3mg/kg intra-muscular DMPA.

**Table 1:** Female macaques: assigned groups and treatment strategies.

Female	Group	Treatment	^*^Measurement
32090	Group 1	Sham	Day 1 (baseline)
32091			
32092			
32093	Group 2	Abrasion (cytobrush)	Day 1 (pre) Day 1 (post)
32094			
32095			
32096	Group 3	N9/N9 (4%, 2 doses, 14hrs apart)	Day 1 (pre) Day 2 (post)
32097			
32098			
32099	Group 4	DMPA (IM, 3mg/kg)	Day 1 (pre) Day 28 (post)
32100			
32101			

*Measurements included: Visual colposcopic examination, OCT imaging, vaginal biopsies.

### Physical Examinations

Twenty-one days prior to the study, vaginal length and width were measured for each female macaque using a malleable uterus sound, which allowed measurement of the vaginal cavity without damaging the mucosa. For examinations, all animals were sedated using 5mg/kg telozole with supplementation of 1-2% isoflurane to oxygen titrated to effect.

During the study, the macaques were examined in the knee-chest position and evaluated with colposcopy followed by OCT imaging (14). The control group was examined once for observations and measurements at baseline. The cytobrush group was examined at baseline prior to use of the cytobrush, then mechanical abrasion with cytobrush was performed and the macaques were examined again immediately after the abrasion was made. The N9 group was examined at baseline prior to the use of N9, then treated with 1.25 ml of 4% N9 per vagina twice (14 hours apart), and examined again 4-6 hours after the second dose was given. The DMPA group was examined at baseline prior to administration of DMPA, then given 3mg/kg IM DMPA, and examined again 28 days later.

### Colposcopy

Colposcopy was performed using a Seiler 525 colposcope with charge-coupled device (CCD) camera attachment. A nasal speculum was used to retract the vaginal tissue and allow for visualization of the vagina and cervix. Digital photographs of the cervix and vagina were obtained. Evaluation of the vagina and cervix was performed using the WHO criteria for colposcopy to evaluate for color changes and epithelial and vascular disruption (19). In addition to determining if disruption or bleeding was present, colposcopy was also used for OCT guidance.

### Optical Coherence Tomography

OCT imaging was performed using a Nirus 1300nm system (Imalux, Cleveland, OH) under colposcopic guidance. A 2.8 mm endoscopic probe was placed in direct contact with the mucosa at the anatomic sites of the cervix, fornices (cervicovaginal junction), and mid-vagina to obtain images measuring 2mm wide and up to 1.8 cm deep. The resolution was 15-25 micrometers, allowing for visualization of the epithelium and submucosa. Three images were obtained at each anatomic site in each of four quadrants (12, 3, 6, and 9 o’clock) for a total of 36 images per animal. The 12 o’clock position corresponded to the posterior vagina and cervix, with 3 o’clock corresponding to the right, 6 o’clock corresponding to the anterior, and 9 o’clock corresponding to the left vagina and cervix. OCT images were visualized in real-time on the OCT system monitor and estimations of vaginal thickness could be made using the scale bar located at the side of the image. After each imaging session was complete, the images were exported from the OCT system. After all study visits were completed, Presto 32 software (Optimec Ltd/BioMedTech LLC, Nizhny Novgorod, Russia) was used to measure epithelial thickness in the images. Each image was evaluated by an experienced grader who was blinded as to treatment group. Measurements were taken using the Presto32 software measuring tool from the surface to the base of the epithelium at three locations (left, middle, and right) in each OCT image The quantitative measurements of epithelial thickness were recorded and utilized for comparison using statistical methods.

### Vaginal Biopsies

After OCT imaging, anterior mid-vaginal biopsies were obtained from each macaque with Tischler biopsy forceps and immediately placed in 10% neutral buffered formalin. Tissue was embedded in paraffin, sectioned, and stained with hematoxylin and eosin (H&E). Slides were evaluated using Nikon C2+ Confocal Module microscope. Digital photographs were obtained for comparison with OCT findings.

### Statistics

Data analysis was performed using Excel with Analysis ToolPak (Microsoft Corporation, Seattle, WA) and RealStats (https://www.real-statistics.com/free-download/real-statistics-resource-pack/) add-ins. A p value of <0.05 was considered to be significant. Data was initially analyzed with one way analysis of variance (ANOVA) due to the large data set and similarity between means and medians, however since the data had a non-normal distribution determined by the Shapiro-Wilk test, Kruskal-Wallis with Nemenyi’s post-hoc test for multiple comparisons was also applied to validate the results. The Kruskal-Wallis and ANOVA results were similar, therefore means and standard deviations were utilized in the graphs presented herein. Macaque age, weight, vaginal length, and vaginal width were reported as median and mean (average) with standard deviation. Epithelial thickness measurements were analyzed in the following manner. Baseline vaginal, fornix, and cervical epithelial thickness measures from all macaques were compared to each other at baseline to determine variations within the anatomic sites. Comparisons of vaginal fornix, and cervical ET were also made between nonmensing and mensing macaques. In the cytobrush, N9, and DMPA treatment groups, comparisons of vaginal, fornix, and cervical epithelial thickness were made before and after treatment.

## Results

### Optimization of Imaging for Small Female Macaque

Because of challenges with performing colposcopy in smaller macaques, we determined measures of vaginal length to optimize the speculum used. The macaques in this study weighed 5.3 ± 0.7 kg and the average age was 4.1 ± 0.2 years. The vaginal length ranged between 3.5-5.0 cm (mean 4.63 ± 0.64) and the width ranged between 0.75-1.0 cm (mean 0.96 ± 0.09) as shown in **Table 2**.

**Table 2:** Female macaques: age, weight, vaginal length and width.

Female (n=12)	*Age (year)	*Weight (kg)	*Length (cm)	*Width (cm)
32090	4.8	6.2	4	1
32091	4.1	5.2	3.5	0.9
32092	4.2	5.6	5	1
32093	4.1	4.5	5	1
32094	4.1	5.1	4	–
32095	4.2	5.9	4.5	–
32096	4.1	5.1	4.75	1
32097	4.0	5.0	4	0.75
32098	4.0	6.9	4.75	1
32099	4.2	5.1	5.5	1
32100	3.8	5.0	5.5	–
32101	4.0	4.3	5	–
*Median*	*4.1*	*5.1*	*4.75*	*1*
*Average*	*4.133*	*5.325*	*4.625*	*0.956*
*STDEV*	*0.2387*	*0.7238*	*0.6351*	*0.0904*

*Pre-study (day: -21).

**Figure 1:**
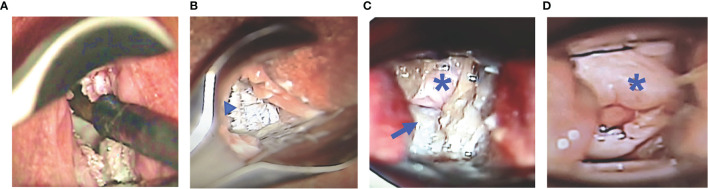
Baseline Colposcopy Findings - use of nasal speculum vaginally for visualization of the cervicovaginal and guidance for optical coherence tomography (OCT) image collection **(A)**. Representative images of the macaque vagina (arrowhead), fornix (long arrow), and cervix (asterisk) viewed through colposcope. The vaginal mucosa is bright white, with pointed finger-like projections and rugations **(B)**, while the cervix is white-pink and smoother **(C)** in a nonmensing macaque. Menstrual blood can be seen at the cervical os, while the cervical mucosa is pink and smooth in a mensing macaque **(D)**.

Both medium (3.5”) and short (2”) nasal specula (Miltex 20-32, 20-28) with locking blades were used for visualization of the cervix, fornices, and midvagina. **Figure 1A** shows the use of a nasal speculum with placement of the OCT probe under direct observation. Due to the pre-set focal length of the colposcope (30 cm), it was not possible to photograph the entire length of the vaginal wall in focus. Images were obtained at the cervix and fornix in a similar plane and then the colposcope was moved slightly back for imaging of the midvaginal wall. The OCT probe was then placed under colposcopic guidance to obtain OCT images at predetermined locations, as described in the methods.

### Baseline Colposcopy Findings

The cervix, fornices, and vagina could be visualized using the colposcope in all macaques, however, it was challenging due to the small size of the vagina. Digital photographs were reviewed at baseline in all 12 macaques. There was variability between animals and at different sites within a single animal. Typically, the vagina had thickened, stiff rugations with variability in height (**Figure 1B**). The fornix typically had a smoother appearance with fewer rugations (**Figure 1C**). The cervix was variable with some macaques having a smooth surface with others having a rough surface (**Figures 1C, D**). In addition, there was great variation in the appearance and quantity of cervical mucus. These findings were most likely related to the phase of the menstrual cycle. Four of the twelve macaques were mensing during the study and tended to have more smooth pink mucosa of the cervix and fornix whereas the mucosa of the other nonmensing macaques was rough and opaque white with pointed finger-like projections.

### Baseline OCT imaging Findings

OCT image findings were similar to that seen in humans (13), pigtailed macaques (12), and sheep (15), except that the epithelial thickness was variable between species. The epithelium could be clearly visualized in real time on the OCT system monitor and in exported images.

**Figure 2:**
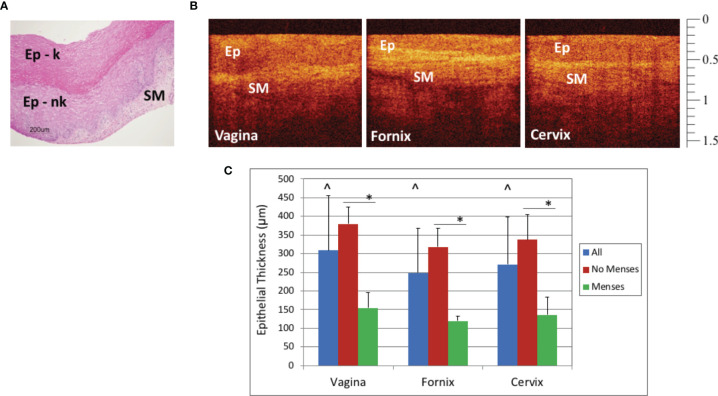
Baseline Optical Coherence Tomography (OCT) and Histology Findings - Representative baseline histology image (hematoxylin and eosin, H&E) of vaginal epithelium with keratinizing (Ep-k) and nonkeratinizing (Ep-nk) epithelium overlying the lamina propria of the submucosa (**A**, scale bar 200 μm) and OCT images from the macaque vagina, fornix, and cervix in which the epithelium (Ep), and the submucosa (SM) can be visualized **B**, OCT calibration bar is in mm, with small tick mark = 100 μm). Baseline epithelial thickness in the vagina, fornix, and cervix was measured by OCT imaging. The vagina was significantly thicker than the fornix (p<0.001) and cervix (p=0.001) and the cervix was significantly thicker than the fornix (p=0.03, significance indicated by ^). Baseline epithelial thickness in the vagina, fornix, and cervix in mensing macaques was thinner when compared to that of non-mensing macaques. (**C**, p < 0.001, significance indicated by *). Error bars represent standard deviation.

Pathology from vaginal biopsies showed that the epithelium had both a thick keratinized non-nucleated superficial layer and a non-keratinized nucleated layer to form the stratified squamous epithelium; H&E was used to confirm OCT image findings. **Figure 2A** shows a representative vaginal biopsy from Group 1, control, with thick keratinized and nonkeratinized layers of epithelium overlying the lamina propria in the submucosal layer. Estimates of OCT thickness could be made in real time using the vertical calibration bar on the side of the image (each small tick mark = 100 μm), however, quantitative measurements for statistical comparisons were made using Presto 32 post-processing software. There was epithelial thickness (ET) variability by site of the image acquisition (e.g. mid-vagina, fornix, cervix) and well as in different macaques.

The ET variability in the site of imaging could be detected by OCT (**Figure 2B**), with the OCT image of the fornix visibly thinner than the OCT image of the vagina. At baseline in these naturally cycling macaques, the mean (± standard deviation) epithelial thickness measured in micrometers was as follows: vaginal ET 308 ± 146, fornix ET 249 ± 118, and cervix 271 ± 128. Vaginal ET was significantly thicker than the fornix (p<0.001) and cervix (p=0.001), while the cervix was significantly thicker than the fornix (p=0.03) (**Figure 2C**).

There was also variability between animals at the baseline assessment. The menstrual cycles of the macaques in this study were not synchronized, and the stage of cycle could not be determined for most of the animals; however, the four animals that were mensing had thinner vaginal epithelium than those that were not mensing (p<0.001, **Figure 2C**). The mean (± standard deviation) epithelial thickness measured in micrometers for nonmensing and mensing macaques was as follows: nonmensing vaginal ET 380 ± 116, fornix ET 312 ± 187, and cervix 338 ± 98 and mensing macaques had vaginal ET 157 ± 66, fornix ET 121 ± 42, and cervix 137 ± 53.

### Treatment With Mechanical Abrasion

**Figure 3:**
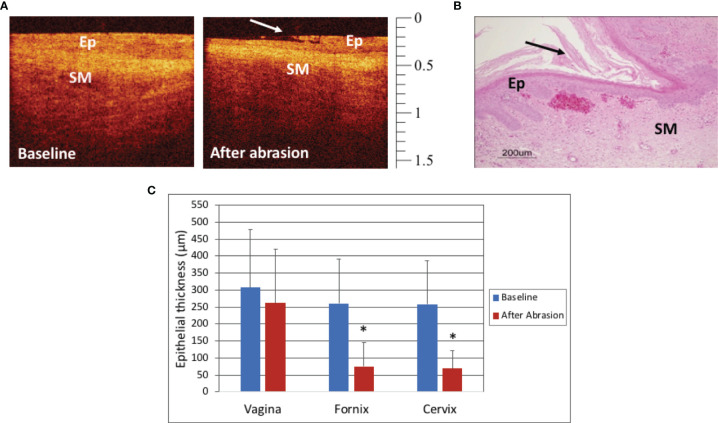
Optical Coherence Tomography (OCT) and Histology Findings after mechanical abrasion - OCT image of the cervix before and after mechanical abrasion **(A)**. The epithelium (Ep) is visibly thinned shortly after the abrasion was performed. The long arrow indicates the edge of epithelial disruption. (OCT calibration bar is in mm, with small tick mark = 100 μm). Hematoxylin and Eosin (H&E) staining of abrasion in the vagina of a mensing macaque (**B**, scale bar 200 μm). Epithelial thickness (ET) measures of the vagina, fornix, and cervix before and after mechanical abrasion showed that ET in the fornices and cervix was thinned after abrasion (**C**, p<0.001), however there was no significant change in vaginal ET (p=0.91). Error bars represent standard deviation and asterisks (*) indicate significance.

The macaques in Group 2 that were treated with mechanical cytobrush abrasion changes seen in real time immediately after the abrasion by both colposcopy and OCT imaging. In the fornix, erythema and occasional bleeding were apparent using colposcopy while minimal changes were visible in the thicker mid-vagina. The extent of epithelial thinning measured by OCT after the abrasions was variable between macaques, and anatomic sites and even within a single OCT image. **Figure 3A** shows OCT images of the fornix at baseline (before) and after the abrasion, where the top layer of the epithelium can be seen to be peeling off. A post-abrasion biopsy histograph shows mucosa with some loss of the superficial epithelium (**Figure 3B**). Quantitatively, the mid-vagina showed no significant change in epithelial thickness (p=0.91), while the vaginal fornices and cervix were significantly thinned after abrasion (p<0.001, **Figure 3C**).

### Treatment With N9

**Figure 4:**
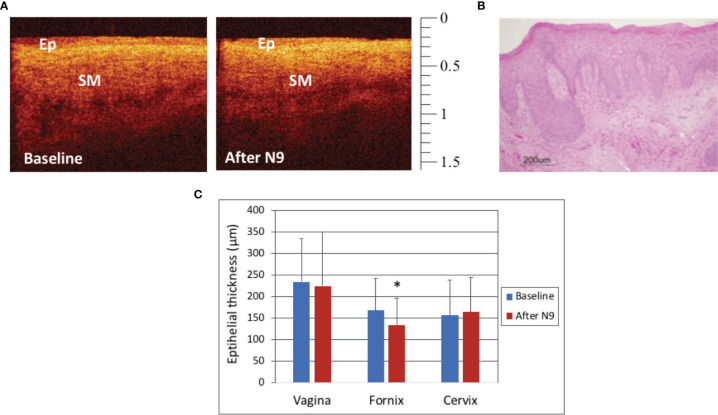
Optical Coherence Tomography (OCT) and Histology Findings after 2 doses of Nonoxynol-9 (N9, 14 hours apart) - OCT image of the fornix in a mensing macaque before and after N9 application **(A)**. The epithelium (Ep) had very subtle changes with slight thinning in the fornix noted after N9. (OCT calibration bar is in mm, with small tick mark = 100 μm). Hematoxylin and Eosin (H&E) staining of vagina of a mensing macaque after N9 (**B**, scale bar 200 μm). Epithelial thickness before and after 2 doses of N9 (14 hours apart) was significantly thinned in the fornix (**C**, p=0.031), however there were no significant changes in the vagina (p=0.51) or cervix (p=0.87). Error bars represent standard deviation and asterisk (*) indicates significance.

The macaques in Group 3 were treated with two doses of N9, 14 hours apart, in order to optimize the treatment effect for access to the basal layer. In this study, there were no visual differences on colposcopy when comparing findings at baseline and after treatment with N9. In most OCT images, no visual differences were visualized, however there were subtle differences noted in some images of the fornix (**Figure 4A**). A representative vaginal histograph from a mensing macaque is shown in **Figure 4B**. **Figure 4C** shows that there were no significant effects on the vaginal and cervical epithelium after N9 treatment (p=0.51, p=0.87, respectively), however the fornix had statistically significant thinning after two applications of N9 (p=0.03). Individual macaques had variable responses, likely due to cycling differences and biological variation.

### Treatment With DMPA

**Figure 5:**
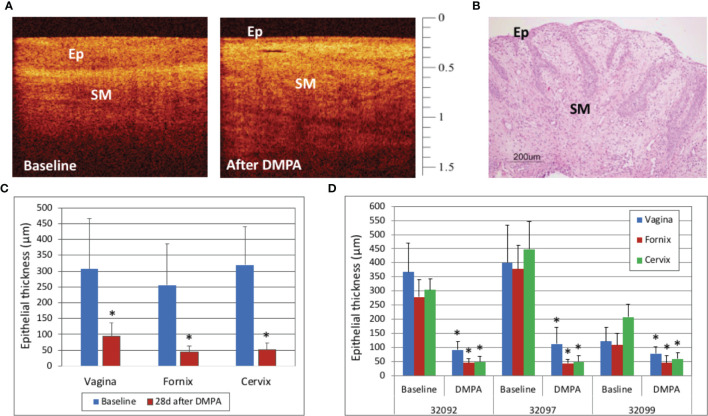
Optical Coherence Tomography (OCT) and Histology Findings after Depomedroxyprogesterone Acetate (DMPA) Treatment – Vaginal epithelial thinning after DMPA could be visualized with OCT imaging (**A**, OCT calibration bar is in mm, with small tick mark = 100 μm) as well as hematoxylin and Eosin (H&E) staining from a vaginal biopsy (**B**, H&E scale bar = 200 μm). Cervicovaginal epithelial thickness measured by OCT before and 28 days after DMPA treatment showed that DMPA significantly thinned epithelium at all anatomic sites (**C**, p<0.001), and while there was variability between animals at baseline, the epithelial thickness (ET) was decreased at all sites in all animals after DMPA (**D**, p<0.001 for all comparisons). Error bars indicate standard deviation and asterisks (*) indicate significance.

In this study, the vaginal, fornix, and cervix, fornix, and epithelium were all thinned 28 days after treatment with DMPA when compared to baseline values (p<0.001), as visualized by OCT and H&E (**Figures 5A–C**). There was variability in the ET at baseline, with one macaque having menses, however all sites in all macaques were thinned after DMPA treatment (p<0.001 for all comparisons, **Figure 5D**).

## Discussion

Macaque cervicovaginal epithelial thickness (ET) was greatest at the mid-vagina and thinnest at the vaginal fornices as observed by optical coherence tomography (OCT) imaging. Mechanical, chemical, and hormonal methods can reduce vaginal ET, however they have potential to affect vaccine efficacy through epithelial disruption or immunosuppression. ET decreased naturally during menses, which may provide an ideal opportunity for accessing the targeted vaginal mucosal basal layers to achieve the optimum ET for intravaginal mucosal vaccination.

Creating a consistent protocol for accessing the epithelial basal cell layers for use in either virus challenge or vaccine administration *via* epithelial thinning can be achieved by various means and quantitated noninvasively by OCT. In this study, we overcame the challenge of visualization of the small rhesus macaque vagina (length 4.6 by 0.96 cm width) by use of a locking nasal speculum and high resolution imaging methods (20). We noted natural variations between animals, during menses, and within various sites (i.e. mid-vagina, vaginal fornices, and cervix) in the cervicovaginal epithelium in macaques. OCT provided real-time estimates of ET, which allowed visualization of treatment effects immediately after intervention, especially after mechanical abrasion. OCT provides the ability to target the site of treatment with a mucosal vaccine based on the morphology of the epithelium as well as to evaluate safety of vaginally applied products during drug development.

The three methods utilized to induce thinning of epithelium (mechanical abrasion with cytobrush, chemical treatment with N9, and hormonal treatment with DMPA) resulted in variable effects. The mechanical abrasion significantly denuded the epithelium of the cervix and fornix, however the thick vaginal epithelium was not consistently affected. When we continued to abrade, the thinnest areas bled. For a mucosal vaccine or efficacy study for STI prevention medications, the epithelium should be thinned but still intact; bleeding areas would compromise such studies because the disrupted areas could allow bypass of the natural mucosal barrier. Previous studies suggest that N9 treatment is less effective to perturb the integrity of the epithelium in NHP when compared to sheep or mice (14, 18, 21). Significant thinning was reported after a single intravaginal dose of N9 in mice or sheep but not in pigtailed macaques (22). Two doses of N9 caused significant thinning of the fornix only; N9 has been shown to increase the likelihood of STI acquisition in women which is a limitation for its use beyond preclinical studies. In this study, DMPA effectively thinned the epithelium, as has been shown after treatment with other progestogens (23), however there is concern that the immunosuppressive effect of DMPA would diminish response to vaccine (24, 25). High progestogen states can suppress cellular immune responses (26), which is a concern for mucosal vaccination, while conversely, estrogen has been shown to improve vaccine uptake (27). While the use of these mechanical, chemical, and hormonal methods may be effective at bypassing the natural barrier of the cervicovaginal epithelium, these methods may also lead to disrupted epithelium and an increased susceptibility to infection. Such methods may not be suitable for vaccine strategies that rely on the integrity of the mucosal epithelium.

The macaques who were naturally on menses during the study had thinner vaginal epithelial thickness measures when compared to nonmensing macaques. We did not obtain longitudinal information on the phase of menstrual cycle in this short cross-sectional study, however a longitudinal study is warranted to further characterize the changes visualized by OCT and colposcopy in the late luteal phase and menses. The finding of thinned vaginal epithelium during menses is consistent with lower estrogen and progesterone levels that occur in the late luteal phase prior to menses (28). Cyclic thinning has been correlated with susceptibility to simian-human immunodeficiency (SHIV) virus infection in pigtailed macaques (*macaca nemestrina*) (29); a thinner superficial cornified layer corresponded to the time of highest likelihood of SHIV infection. In women, epithelial thinning likely plays a role in HIV transmission (8). Genital ulcerative disease, which is characterized by disruption in the protective vaginal epithelial layer, has been shown to increase HIV susceptibility (30). Additionally, women who have used DMPA have an increased susceptibility to HIV, although the mechanism has not been fully elucidated (31). Some studies in women have shown a decrease in vaginal epithelial thickness after use of DMPA and in the luteal phase when compared to the follicular phase (32), albeit to a lesser degree than the ET variations in macaques. Therefore, detection of epithelial thinning can be important in clinical and research settings for STI and HIV prevention strategies.

The fornix was the thinnest site at baseline and the most sensitive site to the methods used to induce thinning. The fornix was thinned after all three treatments, while the cervix was thinned after abrasion and DMPA and the vagina was only thinned after DMPA. This has implications for evaluation of vaginal products undergoing drug and device development during safety evaluations. The fornix is an ideal location for vaccine placement, as the vaccine can be placed into the fornices under direct visualization with a speculum or placed blindly by using tactile perception while using a vaginal catheter for placement. If the finding of thinned epithelium in the vaginal fornices holds true in women, this can have implications for women’s health beyond concerns related to increase in the transmission of STIs and HIV. After removal of the cervix from the vagina during hysterectomy, the vaginal tissue used for closure of the surgical site is comprised solely of the mucosa of the vaginal fornices; this could lead to vaginal cuff dehiscence or surgical site infection, as the mucosal barrier provided by the thinner tissue is weakened. For vaginal mesh placement that extends to the fornices, the thinned epithelium in this location has potential to lead to post-operative complications such as infection, mesh migration, or mesh erosion.

The findings reported in this study have implications for the development of intravaginal mucosal vaccines that rely on incorporation of viral vectors into epithelium stem cells to produce immune responses and propagate within the vagina. Since these types of vaccines will be developed and tested in preclinical models prior to human use, finding a method to optimize timing and location of vaccine delivery early in the development process is crucial to dosing and formulation of a successful mucosal vaccine. Since we determined that the optimal timing for vaccination is likely to occur during menses, the same strategy used in macaque preclinical testing can be used in women without the additional risk of epithelial disruption or increased susceptibility that could be increased by using mechanical, chemical, or hormonal means for epithelial thinning. Furthermore, if the finding that the thinnest area in the vagina in macaques is the fornices holds true in women, the same location can be used for vaccine delivery as well. Studies are currently ongoing to determine if there are site-related differences within the vagina in women.

This goal of this project was to facilitate cervicovaginal epithelial thinning to provide access to the basal cell layer for successful intravaginal mucosal vaccine delivery. Because of limitations with the use of mechanical abrasion, N9, and DMPA due to inconsistent thinning, epithelial disruption, and immune suppression, we hypothesize that menses is likely to be the best strategy for epithelial thinning to provide basal layer access to the vaccine. The next steps include testing that hypothesis by tracking the menstrual cycle in macaques during intravaginal mucosal vaccine studies, introducing the mucosal vaccine into the vagina during menses, and following the immune responses to vaccination.

A strength of this study is the use of the Rhesus macaque model because the selection of a relevant animal model closely related to humans and sensitive to mucosal STI and HIV transmission is crucial. It is well accepted that outbred macaques represent the variability intrinsic to humans as opposed to mice (33–35). The Rhesus macaque model more accurately mimics the human female lower reproductive tract; it is the best characterized animal model for human infectious diseases such as HIV and STIs (9, 17, 33). This animal model recapitulates the essential aspects of pathogenesis and associated immune responses, while permitting invasive studies (36). The Rhesus macaque is also the more recognized animal model to study various candidate microbicides (37).

Additional strengths of the study include the ability to quantitatively monitor multiple sites throughout the cervicovaginal mucosa, which allowed for greater sampling of each macaque when compared to studies limited to the use of biopsies. Additionally, the ability to obtain high resolution images noninvasively and in real time allowed for immediate assessment of the vaginal epithelial thickness in this study; this technology will facilitate real-time observations and interventions in future intravaginal mucosal vaccine delivery studies.

Limitations of the study include the low number of animals per group (three female rhesus macaques per group), the use of young female Rhesus macaques in an early stage of puberty, and the use of various treatments in parallel without pre-selection of the stage of the menstrual cycle. Implications of these limitations include the following. When the number of animals is low, it increases the likelihood of missing a true difference between groups due to insufficient power. This is inherent in the use of nonhuman primate models due to cost, however this study did show thinning after treatment. The use of young animals has the potential for anovulation due to an immature hypothalamic-pituitary-ovarian axis, however the goal of the study did not require ovulatory cycles (38). Finally, the menstrual stage was not synchronized, which led to variations in epithelial thickness between macaques at baseline.

In summary, epithelial thickness and integrity is important to vaginal health and has implications in STI and HIV acquisition as well as intravaginal mucosal vaccine strategies. We utilized various methods to thin the epithelium, however the natural ET decrease during menses may provide an ideal opportunity to access the targeted vaginal mucosal basal layers. Future intravaginal mucosal vaccine studies should include menstrual cycle tracking and evaluation of the immune responses to vaccination during menses.

## Data Availability Statement

The original contributions presented in the study are included in the article/supplementary material. Further inquiries can be directed to the corresponding author.

## Ethics Statement

The animal study was reviewed and approved by The Institutional Animal Care and Use Committee of the Texas Biomedical Research Institute approved the animal protocol (IACUC 1387MM, 04/05/2013).

## Author Contributions

MG and KV contributed to the conception, design, planning and execution of this study as well as data collection and analysis. PAF and ED performed the animal procedures. JW and JY participated in data and image collection and analysis. RW performed lab and data analysis. M-CG and KV analyzed and interpreted the data. M-CG and KV wrote the manuscript. MM contributed to the critical review of the manuscript. All authors contributed to the article and approved the submitted version.

## Funding

This research was funded by the NIH NIAID R56AI084171 (M-CG) and the NIH NIAID R01AI090705 (M-CG) with the support of the Texas Biomedical Research Center and the Southwest National Primate Research Center.

## Conflict of Interest

The authors declare that the research was conducted in the absence of any commercial or financial relationships that could be construed as a potential conflict of interest.
